# Supplementary material for: Evidence That the Protein Phosphatase Activity of PTEN Contributes to Embryonic Development and Tumor Suppression
Source: Cancer Sci. 2026 Jul 19;117(11):2982–96. doi: 10.1111/cas.70476 (PMC13394760; doi:10.1111/cas.70476)
Supplement: Supplementary file 1 [file CAS-117-2982-s001.pdf]

List of supplemetary tables

**Table S1: Antibodies used for western blotting**
**Table S2: Primers used for genotyping mice**
**Table S3: Primers for sequencing *Pten***

**Table S1: Antibodies used for western blotting**

| Antibody | Supplier | Catalogue number | Species reactivity | Dilution |
|---|---|---|---|---|
| PTEN | Santa Cruz Biotechnology | Sc-7974 | Mouse | 1:1000 |
| PTEN | Cell Signaling Technology | 9552 | Rabbit | 1:1000 |
| AKT P-S473 | Cell Signaling Technology | 9271 | Rabbit | 1:1000 |
| AKT P-T308 | Cell Signaling Technology | 9275 | Rabbit | 1:500 |
| AKT | Cell Signaling Technology | 9272 | Rabbit | 1:1000 |
| S6 P-S240/244 | Cell Signaling Technology | 2215 | Rabbit | 1:1000 |
| S6 P-S235/236 | Cell Signaling Technology | 2211 | Rabbit | 1:1000 |
| S6 | Cell Signaling Technology | 2217 | Rabbit | 1:1000 |
| PRAS40 P-T246 | Cell Signaling Technology | 2640 | Rabbit | 1:1000 |
| PRAS40 | Cell Signaling Technology | 2610 | Rabbit | 1:1000 |
| FoxO1 P-T24/FoxO3a P-T32 | Cell Signaling Technology | 9464 | Rabbit | 1:1000 |
| FoxO1 | Cell Signaling Technology | 2880 | Rabbit | 1:1000 |
| GAPDH | Cell Signaling Technology | 2218 | Rabbit | 1:10,000 |

**Table S2: Primers used for genotyping mice**

| Mouse line | Primers for genotyping | Expected size of PCR product |
|---|---|---|
| ***Pten*** $^{+/-}$ | *Pten* common: 5’ TTGCACAGTATCCTTTTGAAG 3’ | Wild-type: 240bp |
| | *Pten* WT: 5’ GTCTCTGGTCCTTACTTCC 3’ | Mutant: 320bp |
| | *Pten* Neo: 5’ ACGAGACTAGTGAGACGTGC 3’ | |
| ***Pten*** $^{+/Y138L}$ | PTEN-Y138L F: 5'-ATGGAAAGGAGTAAATGGATGG-3' | Wild-type: 250bp |
| | PTEN Y138L R: 5'-GGAGTAAAAGCAGGAGAATTGG-3' | Mutant: 300bp |
| ***Pten*** $^{+/Hyp}$ | PTEN 5’ Hypomorph: 5'-TGTTTTTGACCAATTAAAGTAGGCTGTG-3' | Wild-type: 350bp |
| | PTEN 3’ Hypomorph: 5'-AAAAGTTCCCCTGCTGATGATTTGT-3' | Mutant: 490bp |
| ***Pten*** $^{flox/flox}$ | *Pten* Flox F: 5’-GGCAAAGAATCTTGGTGTTAC-3’ | Wild-type: 230bp |
| | *Pten* Flox R: 5’-GCCTTACCTAGTAAAGCAAG-3' | *Pten* $^{flox}$ : 280bp |
| ***Lck-Cre*** | *Lck-Cre* F: 5' CGGTCGATGCAACGAGTGATGAGG 3' | PCR product of transgene at 600bp |
| | *Lck-Cre* R: 5' CCAGAGACGGAAATCCATCGCTCG 3' | |

**Table S3: Primers for sequencing *Pten***

PTEN 5' UTR mouse F : 5'-CATGTTGCAGCAATTCACTG-3'
PTEN 3' UTR mouse R:  5'-GGTATTTTATCCCTCTTGATAAG-3'
