# Supplementary material for: Evidence That the Protein Phosphatase Activity of PTEN Contributes to Embryonic Development and Tumor Suppression
Source: Cancer Sci. 2026 Jul 19:10.1111/cas.70476. Online ahead of print. doi: 10.1111/cas.70476 (PMC13394760; doi:10.1111/cas.70476)
Supplement: Supplementary file 2 — Table S1: Antibodies used for western blotting. Table S2: Primers used for genotyping mice. Table S3: Primers for sequencing Pten. [file CAS-9999-0-s002.pdf]

List of supplemetary tables

Table S1: Antibodies used for western blotting
Table S2: Primers used for genotyping mice
Table S3: Primers for sequencing *Pten*

Table S1: Antibodies used for western blotting

Antibody	Supplier	Catalogue number	Species reactivity	Dilution
PTEN	Santa Cruz Biotechnology	Sc-7974	Mouse	1:1000
PTEN	Cell Signaling Technology	9552	Rabbit	1:1000
AKT P-S473	Cell Signaling Technology	9271	Rabbit	1:1000
AKT P-T308	Cell Signaling Technology	9275	Rabbit	1:500
AKT	Cell Signaling Technology	9272	Rabbit	1:1000
S6 P-S240/244	Cell Signaling Technology	2215	Rabbit	1:1000
S6 P-S235/236	Cell Signaling Technology	2211	Rabbit	1:1000
S6	Cell Signaling Technology	2217	Rabbit	1:1000
PRAS40 P-T246	Cell Signaling Technology	2640	Rabbit	1:1000
PRAS40	Cell Signaling Technology	2610	Rabbit	1:1000
FoxO1 P-T24/FoxO3a P-T32	Cell Signaling Technology	9464	Rabbit	1:1000
FoxO1	Cell Signaling Technology	2880	Rabbit	1:1000
GAPDH	Cell Signaling Technology	2218	Rabbit	1:10,000

Table S2: Primers used for genotyping mice

Mouse line	Primers for genotyping	Expected size of PCR product
Pten $^{+/-}$	*Pten* common: 5’ TTGCACAGTATCCTTTTGAAG 3’	Wild-type: 240bp
	Pten WT: 5’ GTCTCTGGTCCTTACTTCC 3’	Mutant: 320bp
	Pten Neo: 5’ ACGAGACTAGTGAGACGTGC 3’	
Pten $^{+/Y138L}$	PTEN-Y138L F: 5'-ATGGAAAGGAGTAAATGGATGG-3'	Wild-type: 250bp
	PTEN Y138L R: 5'-GGAGTAAAAGCAGGAGAATTGG-3'	Mutant: 300bp
Pten $^{+/Hyp}$	PTEN 5’ Hypomorph: 5'-TGTTTTTGACCAATTAAAGTAGGCTGTG-3'	Wild-type: 350bp
	PTEN 3’ Hypomorph: 5'-AAAAGTTCCCCTGCTGATGATTTGT-3'	Mutant: 490bp
Pten $^{flox/flox}$	*Pten* Flox F: 5’-GGCAAAGAATCTTGGTGTTAC-3’	Wild-type: 230bp
	Pten Flox R: 5’-GCCTTACCTAGTAAAGCAAG-3'	*Pten* ^{flox} : 280bp
Lck-Cre	*Lck-Cre* F: 5' CGGTCGATGCAACGAGTGATGAGG 3'	PCR product of transgene at 600bp
	Lck-Cre R: 5' CCAGAGACGGAAATCCATCGCTCG 3'	

Table S3: Primers for sequencing *Pten*

PTEN 5’ UTR mouse F : 5'-CATGTTGCAGCAATTCACTG-3'
PTEN 3’ UTR mouse R: 5'-GGTATTTTATCCCTCTTGATAAG-3'
